# Bioactive semaphorin 3A promotes sequential formation of sensory nerve and type H vessels during *in situ* osteogenesis

**DOI:** 10.3389/fbioe.2023.1138601

**Published:** 2023-03-06

**Authors:** Xiaoxiao Han, Yuxuan Ma, Weicheng Lu, Jianfei Yan, Wenpin Qin, Jiaying He, Li-Na Niu, Kai Jiao

**Affiliations:** ^1^ The College of Life Science, Northwest University, Xi’an, Shaanxi, China; ^2^ State Key Laboratory of Military Stomatology and National Clinical Research Center for Oral Diseases and Shaanxi Key Laboratory of Stomatology, School of Stomatology, The Fourth Military Medical University, Xi’an, Shaanxi, China

**Keywords:** semaphorin 3A, osteogenesis, nerves, vessels, neurovascular regulators

## Abstract

**Introduction:** Sensory nerves and vessels are critical for skeletal development and regeneration, but crosstalk between neurovascular network and mineralization are not clear. The aim of this study was to explore neurovascular changes and identify bioactive regulators during *in situ* osteogenesis.

**Method:** *In situ* osteogenesis model was performed in male rats following Achilles tenotomy. At 3, 6 and 9 weeks after surgery, mineralization, blood vessels, sensory innervation, and bioactive regulators expression were evaluated *via* micro-computed tomography, immunofluorescent staining, histology and reverse transcriptase-polymerase chain reaction analyses.

**Result:** In the process of *in situ* osteogenesis, the mineral density increased with time, and the locations of minerals, nerves and blood vessels were highly correlated at each time point. The highest density of sensory nerve was observed in the experimental group at the 3rd week, and then gradually decreased with time, but still higher than that in the sham control group. Among many regulatory factors, semaphorin 3A (Sema3A) was highly expressed in experimental model and its expression was temporally sequential and spatially correlated sensory nerve.

**Conclusion:** The present study showes that during *in situ* osteogenesis, innervation and angiogenesis are highly correlated, and Sema3A is associated with the position and expression of the sensory nerve.

## 1 Introduction

Crosstalk between skeletal and neural tissues is critical for skeletal development and regeneration. Previous studies have demonstrated that nerve fibers and bone tissues interact during early embryonic development (Rajpar and Tomlinson, 2022). Increased sensory innervation was confirmed to precede the vascularization, ossification and mineralization of fracture callus (Li et al., 2019). Bone remodeling is regulated by neural signalling under both physiological and pathophysiological conditions (Brazill et al., 2019; Sayilekshmy et al., 2019; Wan et al., 2021). Neuropeptides from sensory nerves, such as substance P (SP) and calcitonin gene-related peptide (CGRP), increase significantly in the bone regeneration area, suggesting that neurological substances play an important role in fracture healing and bone repair (Hofman et al., 2019; Sun et al., 2020). Tropomyosin receptor kinase A-expressing (TrKA-expressing) sensory nerve fibers drive abnormal osteochondral differentiation after soft tissue trauma (Lee et al., 2021). These observations have generated particular interest in sensory nerves related to bone formation.

Vascular network also plays a significant role in bone formation and repair. Blood vessels provide nutritional basis for the surrounding mineralized tissues, whether physiological or pathological (Zhu et al., 2020; Yin et al., 2021). The skeletal system is densely innervated by both neural and vascular networks and neurovascular unit has been identified (Qin et al., 2022a). In addition, innervation has been demonstrated to affect blood vessel assembly and endothelial cell proliferation in bone regeneration (Qin et al., 2022b). However, the role of sensory nerve correlated with vessels during *in situ* osteogenesis and its related mechanisms remain unclear.

Many bioactive regulators are involved in the neurovascular occurrence. Netrins and semaphorins are members of the neuronal guidance cue family (Feinstein and Ramkhelawon, 2017). Accumulating evidence suggests that netrins (Yamagishi et al., 2021) and semaphorins (Avouac et al., 2021; Limoni and Niquille, 2021) are neuronal guidance molecules that facilitate patterning of the nervous and vascular system. However, which bioactive factors influence the neurovascular unit during *in situ* osteogenesis are unknown.

Therefore, the primary aim of the present study was to identify the characteristics of sensory nerves and neovessels during *in situ* osteogenesis, and the secondary aim was to explore the bioactive factors that influence the neurovascular occurrence during bone formation. An *in situ* osteogenesis model was established using rat Achilles tenotomy (Zhang et al., 2020a). Bioactive factors that regulate angiogenesis and sensory innervation during osteogenesis were investigated *via* histology staining, immunofluorescent staining and reverse transcriptase-polymerase chain reaction analyses.

## 2 Materials and methods

### 2.1 Animal experiments

To examine the mechanism of *in situ* osteogenesis, an Achilles tenotomy model was utilized in the present study (EXP). The animal experiments were conducted in accordance with protocols approved by the Institutional Animal Care and Use Committee following the National Institute of Health Guidelines for the Care and Use of Laboratory Animals. All animals were housed in a pathogen-free room and fed with sterilized food and distilled water during the study. No more than 4 animals were housed in a single cage (measuring 50 × 40 × 25 cm) at ambient temperature of 20°C ± 2°C and humidity of 55% ± 5%, with good ventilation and 12-h dark/light cycles (4 W per square meter). Sterilized wood-chip bedding was replaced every other day. Animal health status was monitored twice daily. All animals were healthy from the beginning to the end of the study. No adverse events other than Achilles tendon pathology were observed. A total number of 72 male Sprague-Dawley rats (200–300 g) were randomly divided into following groups with a table of random numbers: (1) Sham; (2) EXP 3-week; (3) EXP 6-week and (4) EXP 9-week. For each animal, four different investigators were involved as follows: a first investigator was responsible for the randomized grouping design. A second investigator performed the surgical procedure, whereas a third investigator identified the characteristics of sensory nerves, vessels and bone formation during *in situ* osteogenesis. Finally, a fourth investigator analyzed data.

Anesthesia was performed by intraperitoneal injection of sodium pentobarbital (40 mg/kg) and pentobarbital overdose were provided to euthanize all rats. The heel of the both hind legs were shaved and sterilized, and then the skin and subcutaneous tissue near the Achilles tendon were incised. This was followed by a transverse disconnection along the midpoint of the Achilles tendon. Both ends of the tendon were clamped repeatedly with vascular clamps about five times and the Achilles tendon was not sutured. In the sham control group, only the skin and subcutaneous tissue were cut to expose the Achilles tendon. At 3, 6 and 9 weeks after surgery, the rats from sham (N = 36) and EXP groups (N = 36) were anesthetized and samples were taken to observe the distribution and content of sensory nerve, type H vessel, and bone. In experimental rats, no differences in bone formation were observed between the left and right legs. Hence, in the first run of the experiment, the left Achilles tendons with calcaneus and lower tibia dissected from rats were fixed with 4% paraformaldehyde for 24 h, then scanned and analyzed with micro-computed tomography (Micro-CT; Inveon, Siemens Preclinical, Knoxville, TN, United States) at high-resolution (N = 6). The right Achilles tendons with calcaneus and lower tibia were embedded in a mixture of methyl methacrylate and dibutyl phthalate and sectioned for hematoxylin-eosin (HE) staining (N = 6). In the second run of the experiment, the ankles with Achilles tendons from the left limbs were dissected, fixed, decalcified and dehydrated with 30% sucrose solution at 4°C for 48 h. Next, the specimens were embedded and processed into 10 μm-thick cryosections for immunofluorescence staining (N = 6). The cartoon-style drawing of section orientation/position was included in Supplementary Figure S1. In addition, the right Achilles tendons tissue from 3-week and 9-week groups were dissected and analyzed by quantitative real-time polymerase chain reaction (RT-PCR), and a single sample was obtained by pooling together every 2 out of 6 Achilles tendons samples (N = 3).

### 2.2 Micro-computed tomography

The Achilles tendons with calcaneus and lower tibia were scanned using micro-computed tomography (Micro-CT; Inveon, Siemens Preclinical, Knoxville, TN, United States) at high-resolution. Briefly, samples were scanned at 80 kV and 500 μA. Two-dimensional slices with 78 μm isotropic resolution were generated. A three-dimensional (3D) image was reconstructed based on the scanned information using the Inveon Research Workplace software (Siemens Medical Solutions United States, Inc., Hoffman Estates, IL, United States). A cylindrical region of interest was positioned over the injury site and the volume of the newly-formed bone was measured by assigning a threshold. Bone mineral density (BMD), bone volume to total volume ratio (BV/TV), and bone surface to bone volume ratio (BS/BV) in the injury were measured using the Inveon Research Acquisition software.

### 2.3 Hematoxylin-eosin staining

The Achilles tendons with calcaneus and lower tibia of sham and experimental rats were excised, fixed in 4% paraformaldehyde for 24 h, dehydrated in ethanol, and embedded in a mixture of methyl methacrylate and dibutyl phthalate. Longitudinal sections (10 µm thickness) were made using a Leica SP1600 hard tissue-slicer. Sections were then used for HE staining. (Wuhan Servicebio Technology Co., Ltd., Wuhan, China).

### 2.4 Immunofluorescence

Specimens were collected from the rats with Achilles tendon injuries in the EXP and sham groups, and the specimens were excised, post-fixed using 4% paraformaldehyde overnight, and decalcified with 10% ethylenediaminetetraacetic acid (EDTA; pH 7.3) for 4 weeks. The demineralization medium was changed every 2 days. After decalcification, the specimens were dehydrated with 30% sucrose solution at 4°C for 48 h. L4 and L5 dorsal root ganglion (DRG) tissues were post-fixed with 1.5% glutaraldehyde for 6 h and cryo-protected with 30% sucrose solution at 4°C for 24 h.

The specimens were embedded in optimal cutting temperature compound (Leica, Germany) and stored at −80°C. Tissue was cut into 5-µm-thin longitudinal sections. The cryofilm (Section-lab, Japan) was mount onto the cut surface, and the specimen was tightly adhered to the cryofilm. Cryosections were stained using standard immunofluorescence methods. Briefly, the sections were permeabilized with 1% Triton X-100 (MilliporeSigma, Burlington, MA, United States) and blocked in 1.5% goat serum (MilliporeSigma, United States). The sections were then incubated overnight with the primary antibodies at 4°C. This was followed by incubation with Alexa Fluor™ fluorescent secondary antibodies (Molecular Probes, United States). All sections were rinsed and mounted with Prolong Diamond Antifade Mountant with 4’,6-diamidino-2-phenylindole (DAPI; Invitrogen, San Diego, CA, United States). The images were captured under a fluorescence microscope (FV1000, Olympus, Tokyo, Japan) and the integrated fluorescence intensity was analyzed using ImageJ software (NIH, Bethesda, MD, United States).

The primary antibodies used were: anti-CGRP antibody (ab36001, Abcam, Cambridge, MA, United States; 14,959, Cell Signaling Technology, Inc. Danvers, MA, United States), anti-PGP9.5 (ab72911, Abcam), anti-platelet and endothelial cell adhesion molecule 1 (PECAM-1, also known as CD31) antibody (sc-376764, Santa Cruz Biotechnology, Inc., Santa Cruz, CA, United States), anti-endomucin (Emcn) antibody (343,158, United States Biological, Salem, MA, United States), and anti-semaphorin 3A (Sema3A) antibody (sc-74555, Santa Cruz Biotechnology). The biological replicates are six because in each group six specimens were embedded and processed for staining and quantification (N = 6). For each sample, three fields of view were selected randomly and the average fluorescence intensity was calculated as the data by Image J software. The control staining without primary or secondary antibody have been performed to confirm the positive staining (Supplementary Figure S2).

### 2.5 Quantitative real-time reverse transcription polymerase chain reaction (qRT-PCR)

To investigate the temporal relationship between sensory nerve and blood vessels during *in situ* osteogenesis, gene expression levels of *Cgrp*, *Sp*, *Cd31*, *Emcn*, *Osx* (osterix), *Ocn*, *Ntn1* (netrin1), *Ntn4* (netrin4), *Sema3a*, and *Sema3e* (semaphorin 3E) of the Achilles tendon tissue in trauma areas were evaluated using qRT-PCR. *Gapdh* (encoding glyceraldehyde-3-phosphate dehydrogenase) was used as the housekeeping gene. Results obtained after calibration using the *Gapdh* expression level were calculated using the 2^−ΔΔCt^ method and presented as fold increases relative to the non-stimulated control (technical replicates *n* = 3 for each group) (Livak and Schmittgen, 2001).

Briefly, total RNA was isolated using the Trizol reagent (Invitrogen). The concentration and purity of the extracted RNA were determined by measuring the absorbance at 260 and 280 nm (BioTek, Winooski, VT, United States). Complementary DNA (cDNA) was synthesized using a PrimeScript RT reagent kit (Takara Bio Inc., Shiga, Japan). The quantitative real-time polymerase chain reaction (qPCR) was performed using the cDNA as the template on a 7,500 Real Time PCR System (Applied Biosystems, Carlsbad, CA, United States). Sense and antisense primers were designed based on the published cDNA sequences using Primer Express 5.0 (Thermo Fisher Scientific, Waltham, MA, United States; Supplementary Table S1).

### 2.6 Statistical analyses

All data are presented as the means ± standard deviations. Data were examined for their normality and homoscedasticity assumptions before the use of parametric statistical methods. Comparisons were analyzed using one-factor analysis of variance (ANOVA) and Tukey’s *post hoc* test. The GraphPad Prism 5 package (GraphPad Software, La Jolla, CA, United States) was employed for the analysis. Statistical significance was preset at α = 0.05. For all charts, groups labeled with different lowercase letters are significantly different (*p* < 0.05).

## 3 Results

### 3.1 Sensory nerve infiltrated around the new bone formation during *in situ* osteogenesis

**FIGURE 1:**
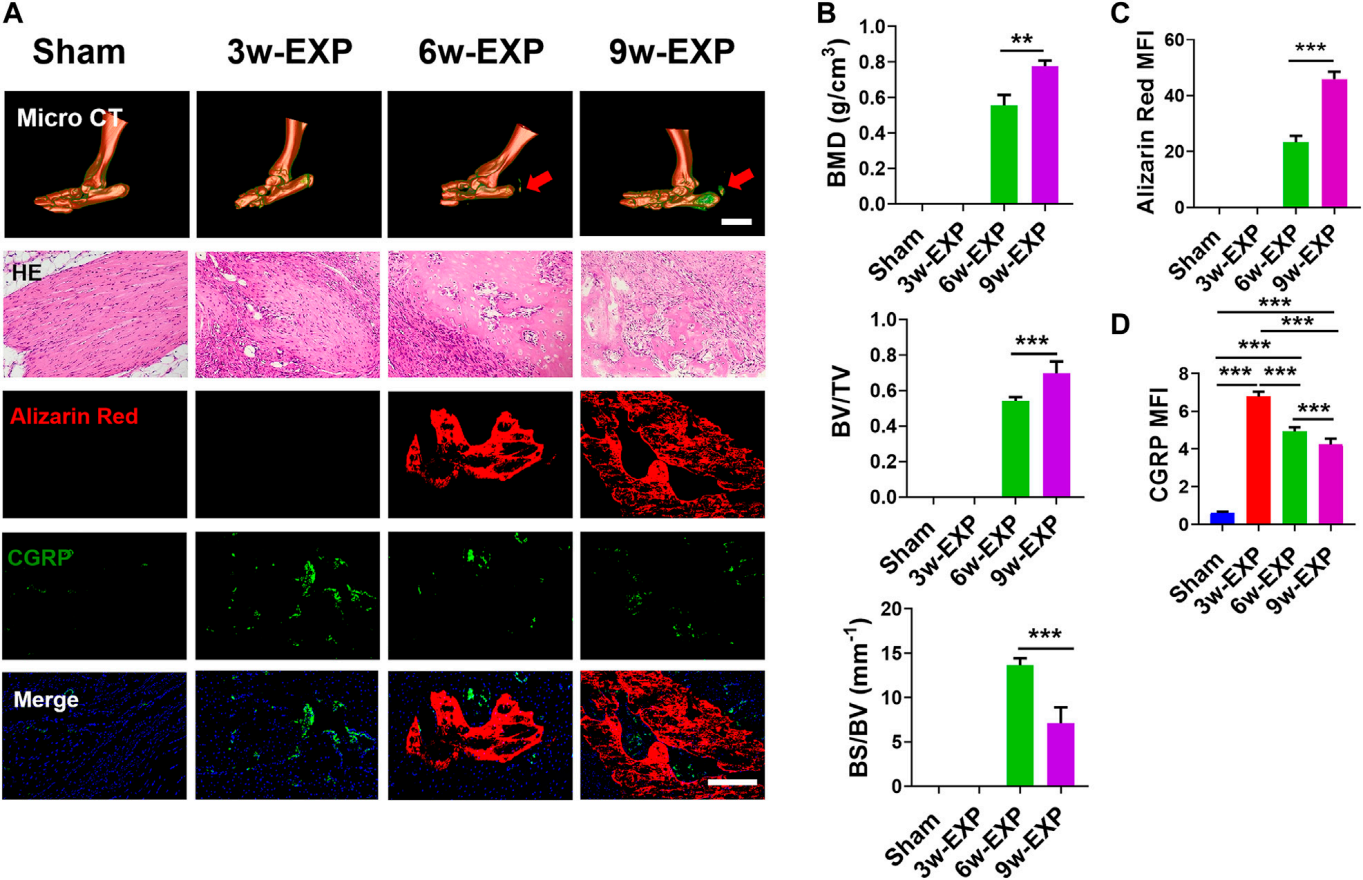
The bone formation and sensory innervation of trauma areas at 3 weeks after a sham operation or 3, 6 and 9 weeks after tenotomy. **(A)** Representative micro-CT images of 3D-reconstructed images of the Achilles tendon injury (bar: 5 mm) and HE, Alizarin Red and immunofluorescence staining of CGRP. Scale bar: 200 μm. **(B)** Quantitative analysis of bone mineral density (BMD), bone volume/total volume (BV/TV), and bone surface/bone volume (BS/BV). **(C)** Quantification of the MFI of Alizarin Red staining per field view. **(D)** Quantification of the MFI of CGRP expression per field view. Nuclei were stained with DAPI (blue). Data represent the means ± standard deviations. For all charts, groups labeled with different lowercase letters are significantly different (*p* < 0.05). CGRP, calcitonin gene-related peptide; DAPI, 4,6-diamidino-2-phenylindole.

The Achilles tendon injury model has been widely used as a model of *in situ* osteogenesis. Micro-CT showed that bone began to form in the 6-week post Achilles tenotomy and continued to enlarge up to 9 weeks, while no obvious calcifications were found in the 3-week EXP group or the sham group. In addition, calcium deposition was evaluated using HE and Alizarin Red staining and the results showed that typical cancellous bone with marrow was noted at 6 and 9 weeks post tenotomy. To clarify the potential relationship between sensory innervation and bone regeneration during bone formation, immunofluorescent staining were conducted to detect calcium deposition and CGRP-positive sensory nerves (Figure 1A). Immunofluorescence staining of protein gene product 9.5 (PGP9.5) also was conducted to examine the presence and distribution of nerve fibers (Supplementary Figure S3).

Micro-CT analysis showed that the bone mineral density (BMD) and bone volume to total volume ratio (BV/TV) in the 9-week EXP group were significantly higher than those of the 6-week EXP group (0.78 ± 0.03 vs. 0.56 ± 0.06, *p* < 0.01; and 0.70 ± 0.07 vs. 0.54 ± 0.02, *p* < 0.01, respectively) (Figure 1B). Conversely, the bone surface to bone volume ratio (BS/BV) in the 9-week EXP group was significantly reduced compared with that in the 6-week EXP group (7.11 ± 1.80 vs. 13.66 ± 0.78; *p* < 0.01). The results suggest that the Achilles tenotomy model successfully simulated the occurrence and development of bone. Similar to the micro-CT analysis, the mineralization in the 9-week EXP group (45.95 ± 2.56) was significantly higher than that in the 6-week EXP group (23.31 ± 0.25) (Figure 1C). The highest MFI of CGRP and PGP9.5 was identified in the 3-week EXP group compared with that in other groups (all *p* < 0.01) (Figure 1D; Supplementary Figure S3). Notably, CGRP-positive sensory nerves co-localized with mineral deposition in both the 6-week and 9-week EXP groups and their distributions were significantly more extensive than that in the sham group (*p* < 0.01). These data suggested that sensory nerves might play an important role during *in situ* osteogenesis.

### 3.2 Infiltration of sensory nerve was followed by angiogenesis during *in situ* osteogenesis

**FIGURE 2:**
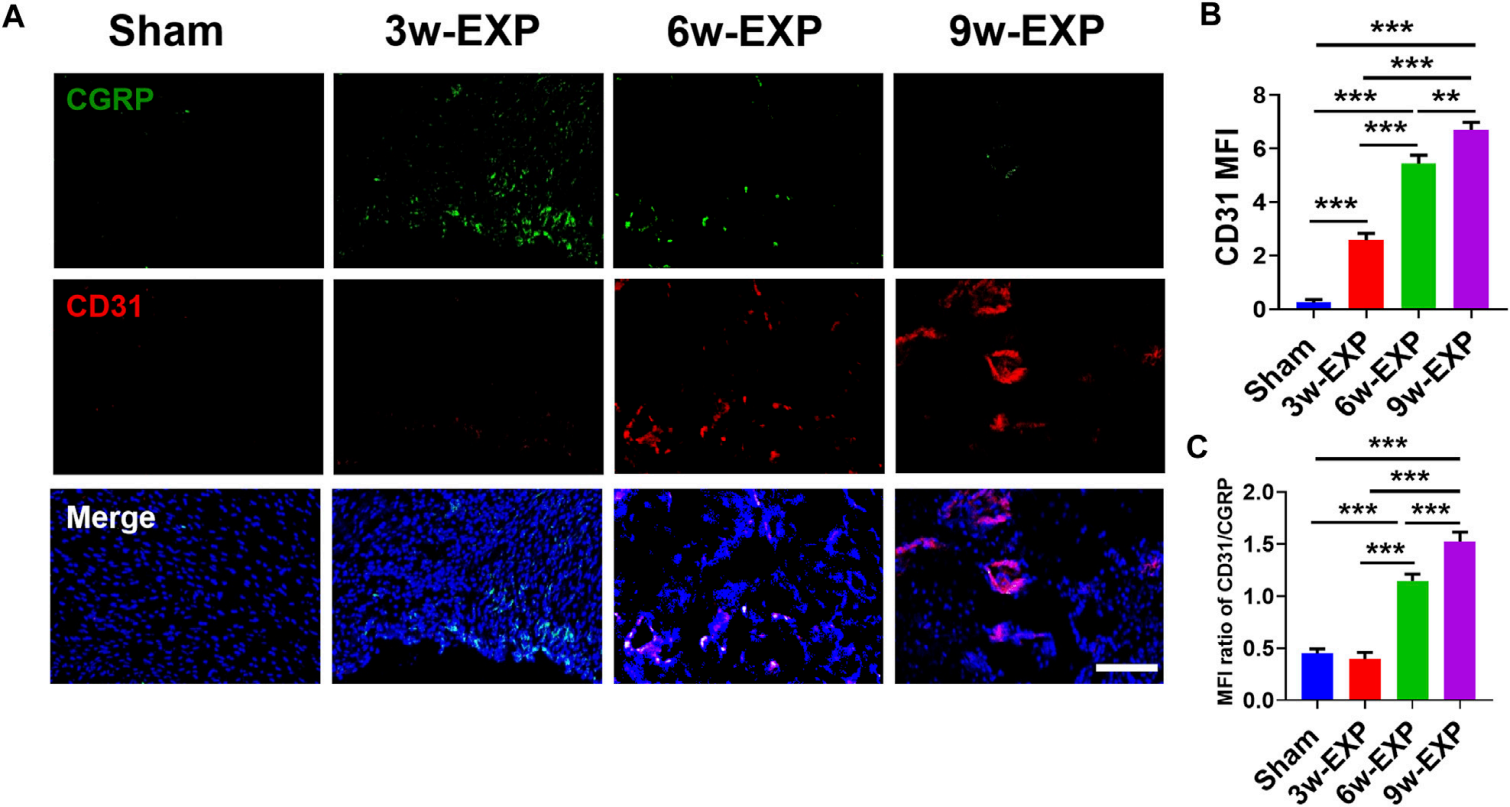
Immunofluorescence staining of trauma areas at 3 weeks after a sham operation or 3, 6 and 9 weeks after tenotomy. **(A)** Representative images of immunofluorescence staining of CGRP and CD31. **(B)** Quantification of the MFI of CD31 expression per field view. **(C)** Quantification of the MFI ratio of CD31/CGRP expression. Nuclei were stained with DAPI (blue). Scale bar: 100 μm. Data represent the means ± standard deviations. For all charts, groups labeled with different lowercase letters are significantly different (*p* < 0.05). CGRP, calcitonin gene-related peptide; DAPI, 4,6-diamidino-2-phenylindole.

**FIGURE 3:**
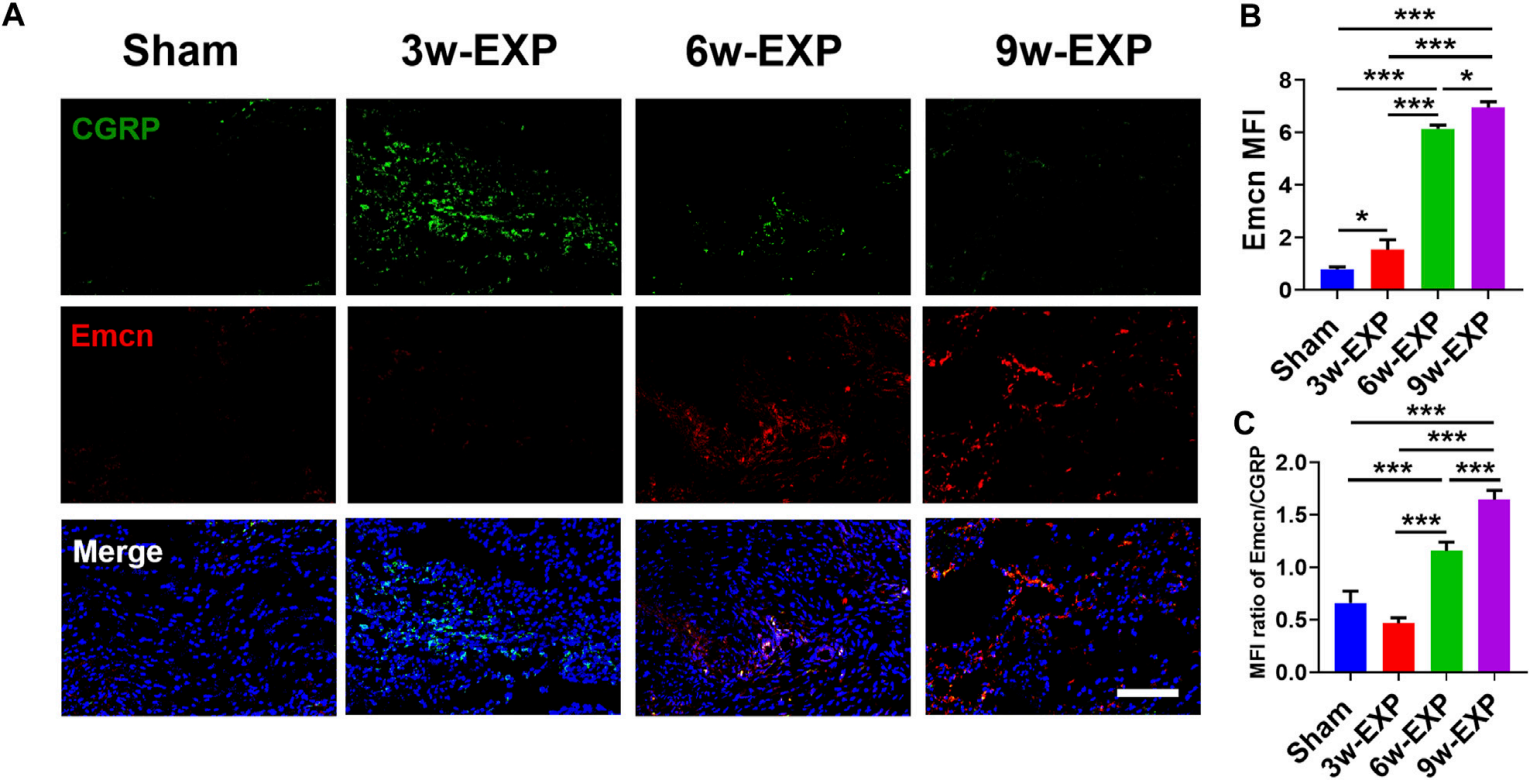
Immunofluorescence staining of trauma areas at 3 weeks after a sham operation or 3, 6 and 9 weeks after tenotomy. **(A)** Representative images of immunofluorescence staining of CGRP and Emcn. **(B)** Quantification of the MFI of Emcn expression per field view. **(C)** Quantification of the MFI ratio of Emcn/CGRP expression. Nuclei were stained with DAPI (blue). Scale bar: 100 μm. Data represent the means ± standard deviations. For all charts, groups labeled with different lowercase letters are significantly different (*p* < 0.05). CGRP, calcitonin gene-related peptide; Emcn, endomucin; DAPI, 4,6-diamidino-2-phenylindole.

Bone-associated nerves and blood vessels are thought to influence the propagation of one another as they grow within bone tissues. To investigate the potential relationship between sensory innervation and angiogenesis, immunofluorescence staining was conducted to show that CD31^+^ and Emcn^+^ vessels co-localized with CGRP^+^ sensory nerves in areas of new bone formation (Figures 2A, 3A). The levels of CD31 and Emcn increased gradually with time (Figures 2B, 3B). Compared with the sham group, the MFI of CD31 and Emcn in the trauma areas increased significantly at 3 and 6 weeks (all *p* < 0.01), and reached the highest value at 9 weeks (*p* < 0.01). Co-staining of CD31 and Emcn representing type H vessels also increased gradually with time and reached the highest value at 9 weeks (all *p* < 0.05) (Supplementary Figure S4). In addition, the MFI ratios of CD31/CGRP and Emcn/CGRP showed no significant differences between the 3-week EXP group and the sham group (all *p* > 0.05), but gradually increased in the 6-week EXP group, reaching their highest levels in the 9-week EXP group, respectively, compared with those of the sham controls (Figures 2C, 3C; all *p* < 0.01).

### 3.3 Gene expression of factors was related to the infiltration of sensory nerves, type H vessels and osteogenesis during *in situ* osteogenesis

**FIGURE 4:**
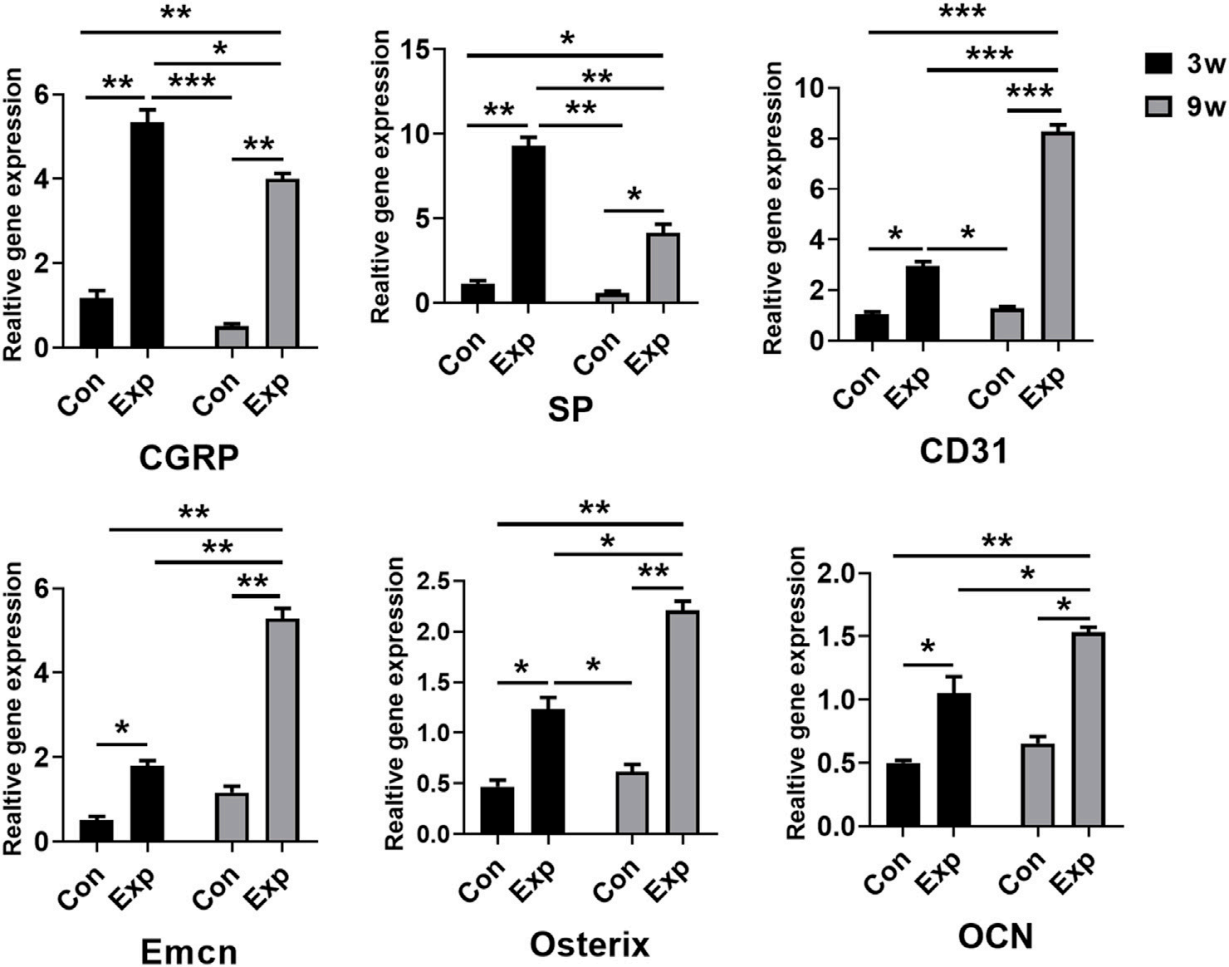
qRT-PCR analysis of genes expressed in the trauma areas after a sham operation or tenotomy at 3 and 9 weeks. For all charts, groups labeled with different lowercase letters are significantly different (*p* < 0.05). CGRP, calcitonin gene-related peptide; SP, substance P; Emcn, endomucin; OCN, osteocalcin.

To confirm the formation of sensory nerves and type H vessels during *in situ* osteogenesis, qRT-PCR was used to detect the expression levels genes related to above processes in the trauma areas at different time points (Figure 4). The mRNA expression levels of sensory nerve-related genes (*Cgrp* and *Sp*), type H vessel-related genes (*Cd31* and *Emcn*), and bone formation related genes (*Osx* and *Ocn*) in the 3-week and 9-week EXP groups were increased significantly compared with those in time-matched sham controls (all *p* < 0.05). In accordance with the immunofluorescent results of the occurring features of sensory innervation and angiogenesis, the expression levels of *Cgrp* and *Sp* gradually decrease from the 3-week EXP group to the 9-week EXP group, while the levels of vessel and bone-related genes showed their higher expression in the 9-week EXP group compared with that in the 3-week EXP group (all *p* < 0.05). By comparison, no significant difference was observed between 3-week and 9-week sham control groups for all target genes (all *p* > 0.05).

### 3.4 Gene expression of factors was related to the infiltration of sensory nerves, type H vessels and osteogenesis during *in situ* osteogenesis

**FIGURE 5:**
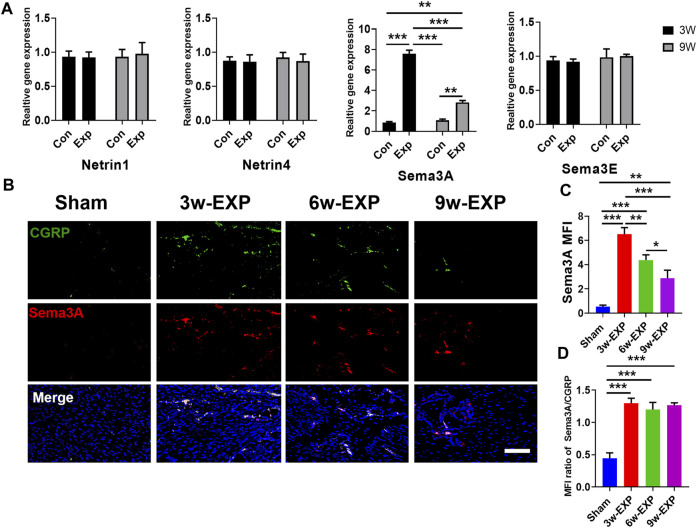
Immunofluorescence staining of trauma areas at 3 weeks after a sham operation or 3, 6 and 9 weeks after tenotomy. **(A)** qRT-PCR analysis of specific genes encoding neuronal guidance molecules in trauma areas in the sham controls and at 3 and 9 weeks in the EXP groups. **(B)** Representative images of immunofluorescence staining of CGRP and Sema3A in the trauma areas in the sham and EXP groups at 3, 6 and 9 weeks. **(C)** Quantification of the MFI of Sema3A expression. **(D)** Quantification of the MFI ratio of Sema3A/CGRP expression. Nuclei were stained with DAPI (blue). Scale bar: 100 μm. Data represent the means ± standard deviations. For all charts, groups labeled with different lowercase letters are significantly different (*p* < 0.05). Sema3A, semaphorin 3A; Sema3E, semaphorin 3E; CGRP, calcitonin gene-related peptide; DAPI, 4,6-diamidino-2-phenylindole.

The immunofluorescence and qRT-PCR results both indicated that sensory nerve innervation occurred earlier than vascularization during *in situ* osteogenesis, we speculated that some neuronal guidance molecules from sensory nerves might induce angiogenesis and osteogenesis in the trauma areas (Figure 5A). Therefore, we detected the mRNA levels of those neuronal guidance molecules that related to angiogenesis and osteogenesis, such as *Ntn1, Ntn4, Sema3a*, and *Sema3e* (Sakurai et al., 2010; Yebra et al., 2011; van Gils et al., 2013). The results showed that the mRNA levels of *Sema3a* were significantly higher in the 3-week EXP group (7.83 ± 0.27) than in the 9-week EXP group (2.83 ± 0.27), and both levels were significantly higher than those in the sham controls (all *p* < 0.05). The cell bodies of afferent nerves are generally positioned in the dorsal root ganglia (DRG). Sema3A expression was significantly higher for the DRG tissues obtained from the 3-week EXP group, compared with other groups (all *p* < 0.05) (Supplementary Figure S5). There was no significant difference in the mRNA levels of *Ntn1, Ntn4*, and *Sema3e* between the 3-week and 9-week EXP groups and the sham controls (all *p* > 0.05). Specimens stained for CGRP and Sema3A enabled simultaneous visualization of the location of sensory nerves and Sema3A expression (Figure 5B). CGRP-positive sensory nerves obviously co-localized with Sema3A in the 3-week and 6-week EXP groups and their distributions were more extensive than those in the sham controls, indicating the neural production of Sema3A in trauma areas. Similar to the CGRP quantification analysis, the highest MFI of Sema3A was identified in the 3-week EXP group (5.72 ± 0.28), compared with that in the 9-week EXP group (2.88 ± 0.66) and sham control groups (0.54 ± 0.12) (Figure 5C). The MFI ratios of Sema3A/CGRP were further used to confirm the presence of neural production of Sema3A in trauma areas. The ratios in all the EXP groups were around 1, and did not exhibit any significant difference among the EXP groups (all *p* > 0.05) (Figure 5D). Taken together, the results support the co-expression and co-localization of Sema3A and CGRP. Therefore, Sema3A might be an important signaling molecule that promotes osteogenesis and angiogenesis during *in situ* osteogenesis.

## 4 Discussion

The present study showed that the highest sensory innervation density was observed in the 3rd week after tendon injury, and then decreased gradually with time. At the same time, angiogenesis and osteogenesis both increased over time until the 9th week. In particular, sensory nerves, blood vessels and ectopic bone showed good spatiotemporal correlations. Furthermore, among many sensory nerve-derived regulators of angiogenesis and osteogenesis, Sema3A was observed to be highly expressed in the bone formation areas and thus might be an important signaling molecule that promotes bone formation. The present data show that sensory nerves and neovessels during *in situ* osteogenesis associates with Sema3A secretion.

In accordance with previous studies, our results showed that tendon injury caused bone formation, and the degree of ossification increased with time. The 9-week EXP group showed the highest BV/TV and BMD. In addition, the 6-week EXP group had a higher BS/BV compared with the 9-week group, which suggested that the new bone formed in the early stage of *in situ* osteogenesis is more interconnected and porous. No values for BV/TV and BMD were obtained for the 3-week EXP group *via* micro-CT analysis, which might have been caused by the level of minerals being too low to reach the scanning threshold. The distribution of either CGRP^+^ or PGP9.5^+^ nerves reached its peak at 3 weeks after tendon injury, and gradually decreased with increased time; however, it remained consistently higher than that in the sham controls. The correspondence between increased sensory innervation and early osteogenesis suggests that sensory nerves play an important role in bone formation, which is consistent with previous studies on embryonic skeleton growth and bone regeneration (Tomlinson et al., 2016; Marrella et al., 2018; Li et al., 2019; Tomlinson et al., 2020). However, male rats were used in the present and previous studies to exclude the effects of estrogen, so the translation of these results to females needs to be verified (Clarke, 2020).

It has been reported that peripheral nerves appear earlier than blood vessels during embryonic skeleton growth (Li et al., 2019). Nerve fibers appear initially in the vicinity of areas with high osteogenic activities and rich capillary networks in the developing skeleton (Tomlinson et al., 2016; Tomlinson et al., 2020). The present results showed that CGRP^+^ sensory nerves were co-localized closely with CD31^+^ or Emcn^+^ blood vessels throughout the whole process of bone formation, and the numbers of CD31^+^ or Emcn^+^ blood vessel cells increased simultaneously until the end of the 9th week after injury. H-type vascular endothelial cells, with high expression of CD31 and Emcn, are tightly related to the function of osteoblastic cells and have a strong ability to induce new bone formation (Zhang et al., 2020b). The MFI ratios of CD31/CGRP and Emcn/CGRP in the 6-week and 9-week EXP groups were both significantly higher than those in the 3-week EXP group. The changes of these ratios reflect the difference in the occurrence time between innervation and angiogenesis dynamically, i.e., sensory nerve fibers participate in the osteogenic activities earlier, while vascularization follows innervation and gradually increases during *in situ* osteogenesis.

Many signaling molecules are involved in the coupling of innervation and angiogenesis. Netrins and semaphorins are known as neuronal guidance molecules that facilitate patterning of the nervous and vascular system. Netrin-1 and Sema3A were demonstrated to repel leukocytes in response to laminar shear stress, thus acting as mediators of adaptation to hemodynamic environment (Yebra et al., 2011). Deprivation of Sema3A adversely affects skeletal vascularization and mineralization (Li et al., 2017; Hayashi et al., 2019). Mice lacking Sema3A in neurons displayed diminished bone mass, sensory innervation and vascularization (Fukuda et al., 2013). The present study found that the Sema3A level increased significantly in the bone formation model and was primarily co-localized with CGRP^+^ nerves which were spatially correlated with the distribution of CD31^+^ and Emcn^+^ blood vessels. These results suggested that vessels maintain their responsiveness to Sema3A spatially and temporally, and the increased distribution of blood vessels during *in situ* osteogenesis may result from the increased expression of Sema3A from sensory nerve. SEMA3 proteins have been demonstrated to control integrin-mediated adhesion, allowing for vascular remodeling (Serini et al., 2003). Particularly, Sema3A can inhibit the binding of VEGF_165_ to Neuropilin-1 (NRP1) and regulate angiogenesis (Neufeld and Kessler, 2008). These findings demonstrate that Sema3A is essential to form the more mature-appearing vascular patterns. To further study the interaction of nerves and vessels during *in situ* osteogenesis, the co-staining and proximity assay of CGRP^+^, CD31^+^Emcn^+^ and NRP1^+^ structures are required.

The present study showed that abundant sensory fibers accompanied by increased Sema3A expression first appeared during *in situ* osteogenesis, followed by vascular formation and bone formation. During *in situ* osteogenesis, innervation and angiogenesis are spatially correlated; Sema3A is highly expressed and associated with the position of the sensory nerve. Based on above results, we proposed that the abnormal sensory innervation was one of the early events of bone formation, and thus targeting the sensory nerve ingrowth or Sema3A may be the potential targets for regulating bone formation.

## Data Availability

The original contributions presented in the study are included in the article/Supplementary Material, further inquiries can be directed to the corresponding author.
