# Supplementary material for: Bioactive semaphorin 3A promotes sequential formation of sensory nerve and type H vessels during in situ osteogenesis
Source: Front Bioeng Biotechnol. 2023 Mar 6;11:1138601. doi: 10.3389/fbioe.2023.1138601 (PMC10025372; doi:10.3389/fbioe.2023.1138601)
Supplement: Supplementary file 1 [file DataSheet1.docx]

Supplementary Material

Bioactive semaphorin 3A promote sequential formation of sensory nerve and type H vessels during in situ osteogenesis

Xiaoxiao Han^#,1,2^, Yuxuan Ma^#,2^, Weicheng Lu^#,2^, Jianfei Yan^2^, Wenpin Qin^2^, Jiaying He^2^, Li-Na Niu^2^, Kai Jiao^*,2^

*** Correspondence:** Kai Jiao: kjiao1@163.com

# Supplementary Figures and Tables

## Supplementary Figures


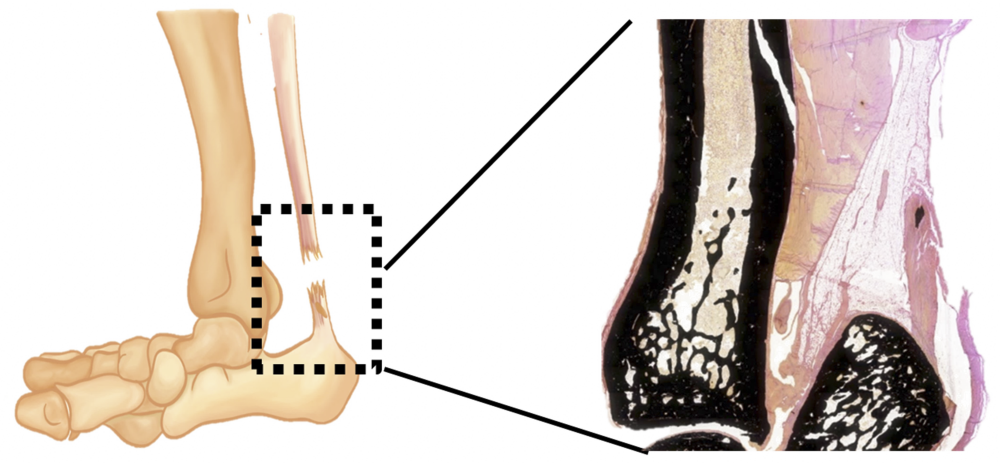


**Supplementary Figure 1.** The cartoon-style drawing of the orientation/position of sections.


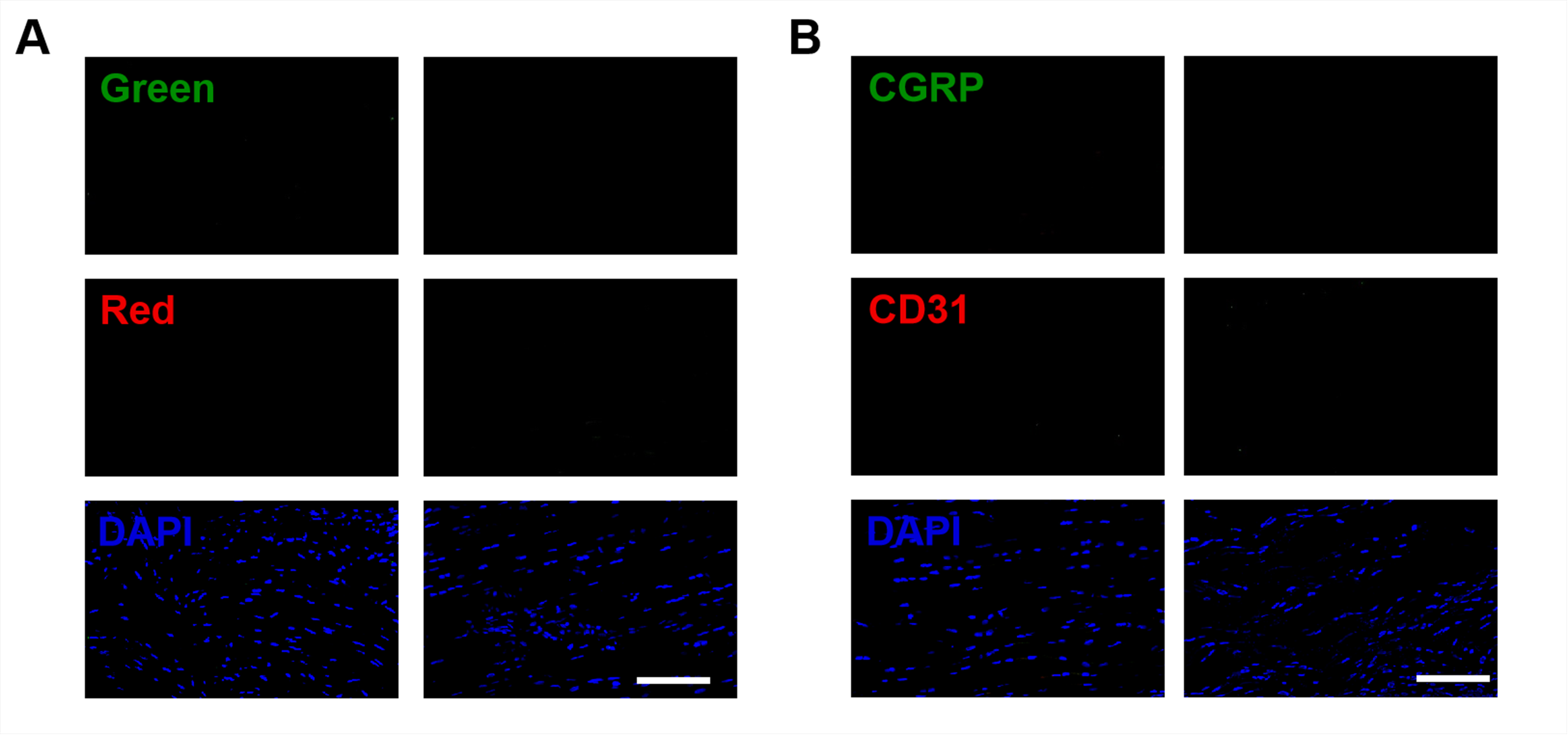


**Supplementary Figure 2.** Immunofluorescence image without primary antibody or secondary antibody. (**A**) Representative images of immunofluorescence staining without primary antibody. (**B**) Representative images of immunofluorescence staining without secondary antibody. Nuclei were stained with DAPI (blue). Scale bar: 100 μm.


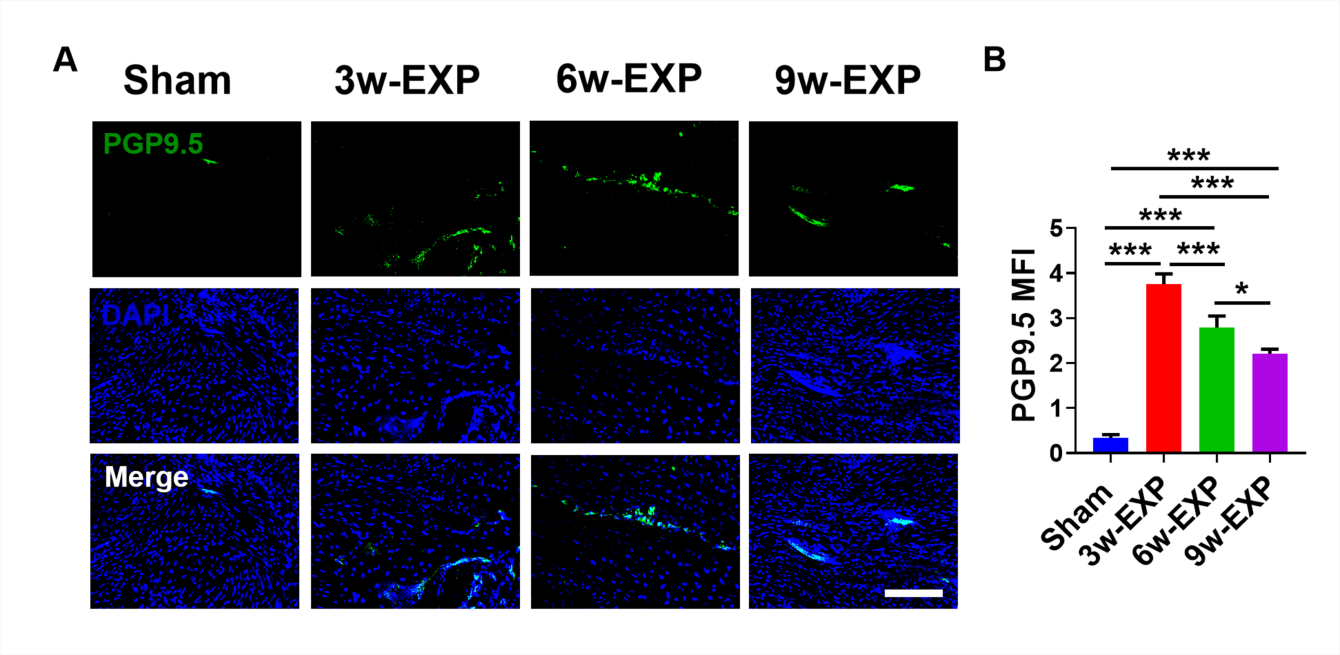


**Supplementary Figure 3.** Representative images of immunofluorescence staining of PGP9.5 staining. (**A**) Representative images of immunofluorescence staining of protein gene product 9.5 (PGP9.5) in the trauma areas in the sham controls (3 weeks) and at 3, 6 and 9 weeks in the EXP groups. (**B**) Quantification of the mean fluorescence intensity (MFI) of PGP9.5 expression per field view. Nuclei were stained with DAPI (blue). Scale bar: 100 μm. Data represent the means ± standard deviations. For all charts, groups labeled with different lowercase letters are significantly different (p < 0.05).


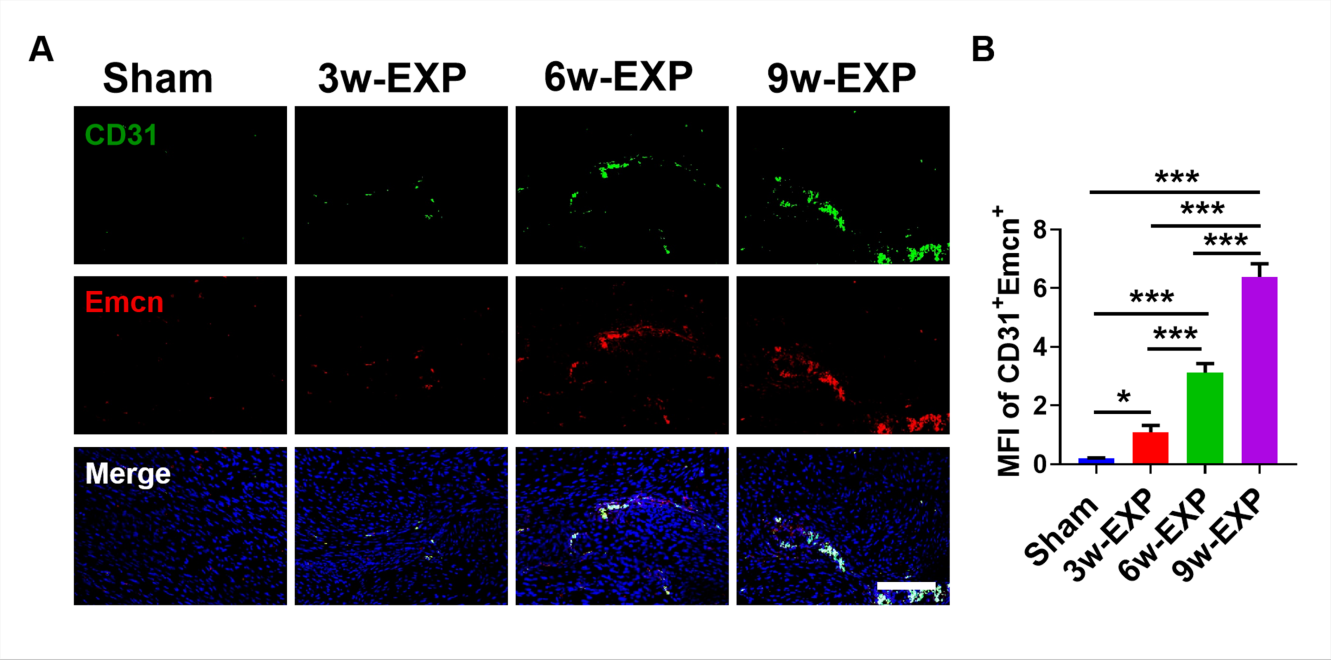


**Supplementary Figure 4.** Representative images and quantification of immunofluorescent co-staining of CD31 and Emcn near to the ectopic bone sites. (**A**) Representative images and (**B**) quantification of immunofluorescent co-staining of CD31 and Emcn near to the ectopic bone sites after a sham operation (3 weeks) or at 3, 6 and 9 weeks after tenotomy. Nuclei were stained with DAPI (blue). Scale bar: 100 μm.


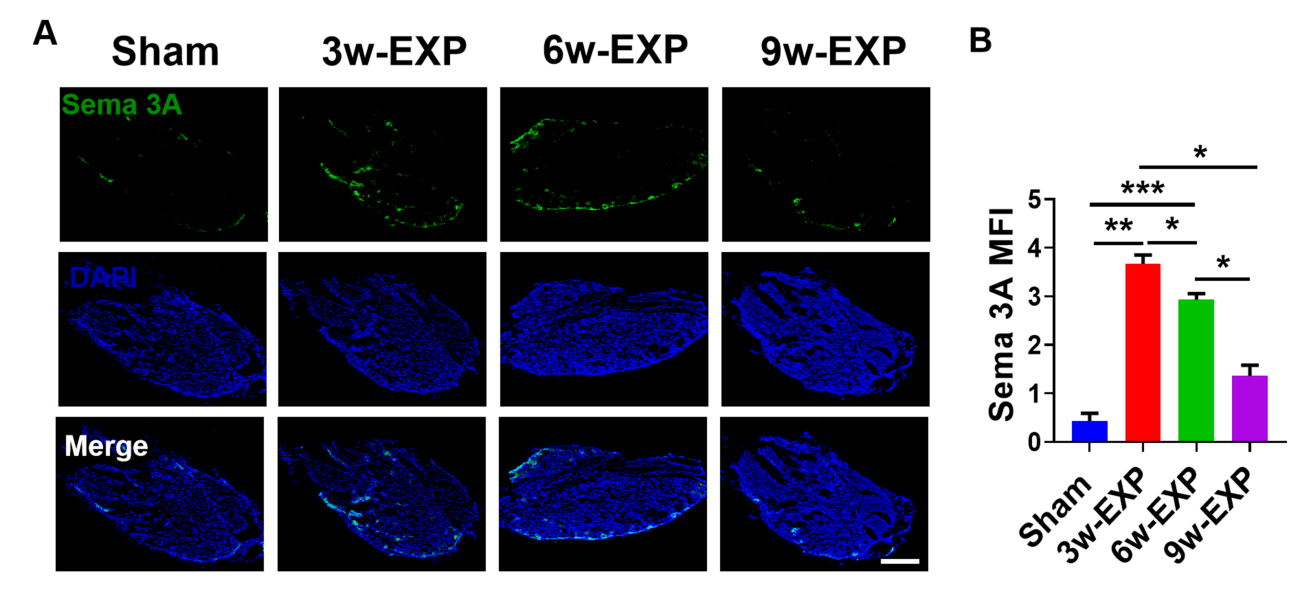


**Supplementary Figure 5.** Representative images of DRG immunostained with Sema3A. (**A**) Representative images of dorsal root ganglia (DRG) immunostained with Sema3A in the sham controls (3 weeks) and at 3, 6 and 9 weeks in the EXP groups. (**B**) Quantification of the mean fluorescence intensity (MFI) of Sema3A expression per field view. Nuclei were stained with DAPI (blue). Scale bar: 200 μm. Data represent the means ± standard deviations. For all charts, groups labeled with different lowercase letters are significantly different (p < 0.05).

## Supplementary Table

| **Gene name** | **Forward/ Reverse** | **Sequence （5’-3’）** |
| --- | --- | --- |
| calcitonin-related polypeptide | Forward | CCTGGTTGTCAGCATCTTGCTC |
|  | Reverse | TGCACCAGTGCAGCCAGTA |
| Substance P (Tachykinin) | Forward | ACTGGTCCGACAGTGACCAAATC |
|  | Reverse | CCCGTTTGCCCATTAATCCA |
| CD31 | Forward | TGGTTGTCATTGGAGTGGTC |
|  | Reverse | TTCTCGCTGTTGGAGTTCAG |
| Emcn | Forward | CCAGTCTTCTCACCAGTCTACAC |
|  | Reverse | TCACAGTTCCTCCTAGCAAAGTC |
| Osterix | Forward | CCTACTTACCCGTCTGACTTTGC |
|  | Reverse | TCCAGTTGCCCACTATTGCC |
| OCN | Forward | TGACAAAGCCTTCATGTCCAA |
|  | Reverse | CTCCAAGTCCATTGTTGAGGTAG |
| Netrin1 | Forward | GTTCGGCGACGAGAACGAA |
|  | Reverse | TGTGCCTACAGTCACACACCAGA |
| Netrin4 | Forward | AGATCACCAACCTCCGAGTGC |
|  | Reverse | GCCCTTGACGATGAAGTCATAGAC |
| Semaphorin 3A | Forward | GGATTTCATGGGACGAGACTTTG |
|  | Reverse | GAGATGGGCACTGATGAATCTAGGA |
| Semaphorin 3E | Forward | GAATCCTGGTGAATAAGTGGAGCA |
|  | Reverse | TGGGATCTCGGGTAGGCAGTA |

**Supplementary Table 1.** Primer sequences used for RT-PCR in the present study.
